# Genetic diversification of *Panstrongylus geniculatus* (Reduviidae: Triatominae) in northern South America

**DOI:** 10.1371/journal.pone.0223963

**Published:** 2019-10-17

**Authors:** Valentina Caicedo-Garzón, Fabian C. Salgado-Roa, Melissa Sánchez-Herrera, Carolina Hernández, Luisa María Arias-Giraldo, Lineth García, Gustavo Vallejo, Omar Cantillo, Catalina Tovar, Joao Aristeu da Rosa, Hernán J. Carrasco, Maikell Segovia, Camilo Salazar, Juan David Ramírez

**Affiliations:** 1 Grupo de Investigaciones Microbiológicas–UR (GIMUR), Departamento de Biología, Facultad de Ciencias Naturales y Matemáticas, Universidad del Rosario, Cra. Bogotá D.C., Colombia; 2 Grupo de Genética Evolutiva, Filogeografía y Ecología de la Biodiversidad Neotropical (GEUR), Departamento de Biología, Facultad de Ciencias Naturales y Matemáticas, Universidad del Rosario, Bogotá D.C., Colombia; 3 Universidad Nacional de San Simón, Cochabamba, Bolivia; 4 Laboratorio de Investigaciones en Parasitología Tropical (LIPT), Universidad del Tolima, Ibagué, Colombia; 5 Laboratorio de Referencia e Investigación en Enfermedades Tropicales, Dirección de Sanidad Ejército, Ejército Nacional de Colombia, Bogotá, Colombia; 6 Grupo de investigación en Enfermedades Tropicales y Resistencia Bacteriana, Programa de Medicina, Facultad de Ciencias de la Salud, Universidad del Sinú, Montería, Colombia; 7 Laboratório de Parasitologia, Departamento de Ciências Biológicas, Faculdade de Ciências Farmacêuticas, Universidade Estadual Paulista “Júlio de Mesquita Filho” (UNESP), Araraquara, SP, Brasil; 8 Laboratorio de Biología Molecular de Protozoarios, Instituto de Medicina Tropical, Universidad Central de Venezuela, Caracas, Venezuela; University of Kentucky, UNITED STATES

## Abstract

Triatomines are the vectors of *Trypanosoma cruzi*, the etiological agent of Chagas disease. Although *Triatoma* and *Rhodnius* are the most-studied vector genera, other triatomines, such as *Panstrongylus*, also transmit *T*. *cruzi*, creating new epidemiological scenarios. *Panstrongylus* has at least 13 reported species but there is limited information about its intraspecific genetic variation and patterns of diversification. Here, we begin to fill this gap by studying populations of *P*. *geniculatus* from Colombia and Venezuela and including other epidemiologically important species from the region. We examined the pattern of diversification of *P*. *geniculatus* in Colombia using mitochondrial and nuclear ribosomal data. Genetic diversity and differentiation were calculated within and among populations of *P*. *geniculatus*. Moreover, we constructed maximum likelihood and Bayesian inference phylogenies and haplotype networks using *P*. *geniculatus* and other species from the genus (*P*. *megistus*, *P*. *lignarius*, *P*. *lutzi*, *P*. *tupynambai*, *P*. *chinai*, *P*. *rufotuberculatus* and *P*. *howardi*). Using a coalescence framework, we also dated the *P*. *geniculatus* lineages. The total evidence tree showed that *P*. *geniculatus* is a monophyletic species, with four clades that are concordant with its geographic distribution and are partly explained by the Andes orogeny. However, other factors, including anthropogenic and eco-epidemiological effects must be investigated to explain the existence of recent geographic *P*. *geniculatus* lineages. The epidemiological dynamics in structured vector populations, such as those found here, warrant further investigation. Extending our knowledge of *P*. *geniculatus* is necessary for the accurate development of effective strategies for the control of Chagas disease vectors.

## Introduction

Chagas disease affects about six million people in Latin America and is caused by the parasite *Trypanosoma cruzi*, which is mainly transmitted by insects of the subfamily Triatominae (Hemiptera: Reduviidae) [1]. The subfamily Triatominae is composed of five tribes, 19 genera, and 154 described species [2,3], but only a few genera are involved in the transmission of *T*. *cruzi* [4,5]. The genera *Triatoma*, *Rhodnius*, and *Panstrongylus* are the main vectors that transmit the parasite to humans [1,6]. After *Triatoma* and *Rhodnius*, *Panstrongylus* is the genus with the most species (currently 13), some of which appear to be involved in a domiciliation process (insofar as at least three developmental life stages can be found in homes) [5]. However, studies of *T*. *cruzi* transmission and control strategies have mainly focused on *Rhodnius* and *Triatoma*, and secondary vectors such as *Panstrongylus* remain unstudied. The species relationships, genetic diversity, and evolutionary trends of this genus are also rarely studied, in contrast to those of *Rhodnius* and *Triatoma*.

The incursion of *P*. *geniculatus* into the home is relevant because of the marked emergence of sylvatic *T*. *cruzi* strains, which are more virulent than domestic strains [7,8]. This change in vector behavior alters the transmission dynamics for *T*. *cruzi* and poses new challenges for the control of Chagas disease [2,5,9]. *Panstrongylus geniculatus* occurs in 18 countries in Latin America, from Mexico to Argentina, with a vast range of habitats, including dry, humid, rainy forest, and savannah environments [5]. This species is one of the principal vectors incriminated in the outbreaks of oral Chagas disease in Colombia and Venezuela [10–12], with reportedly higher parasitemia than other infection routes [2,7,10].

*Panstrongylus geniculatus* has a broad distribution in Colombia [13] where its domiciliation increases the risk of new Chagas disease cases. Previous studies have indicated that *P*. *geniculatus* has the highest frequency of *T*. *cruzi* infection in this country [1]. However, regardless of the epidemiological importance of this species, its current classification is difficult and based exclusively on morphological analyses [5,14–17]. Despite advances in molecular methods, few studies have used DNA markers to evaluate the phylogenetic status of this genus in the subfamily Triatominae, and the species relationships within *Panstrongylus* remain unclear. Previous studies have shown incongruence in the phylogeny of the species, suggesting polyphyly [18] and paraphyly [19]. As far as we know, there is no information about the processes and factors shaping the intraspecific and interspecific diversity in this genus. Here, we begin to address this lack of information using molecular data to document the genetic diversity of *P*. *geniculatus*. Our results extend our knowledge of this vector and our understanding of the biology of *P*. *geniculatus*.

## Methods

### Sampling

**Fig 1:**
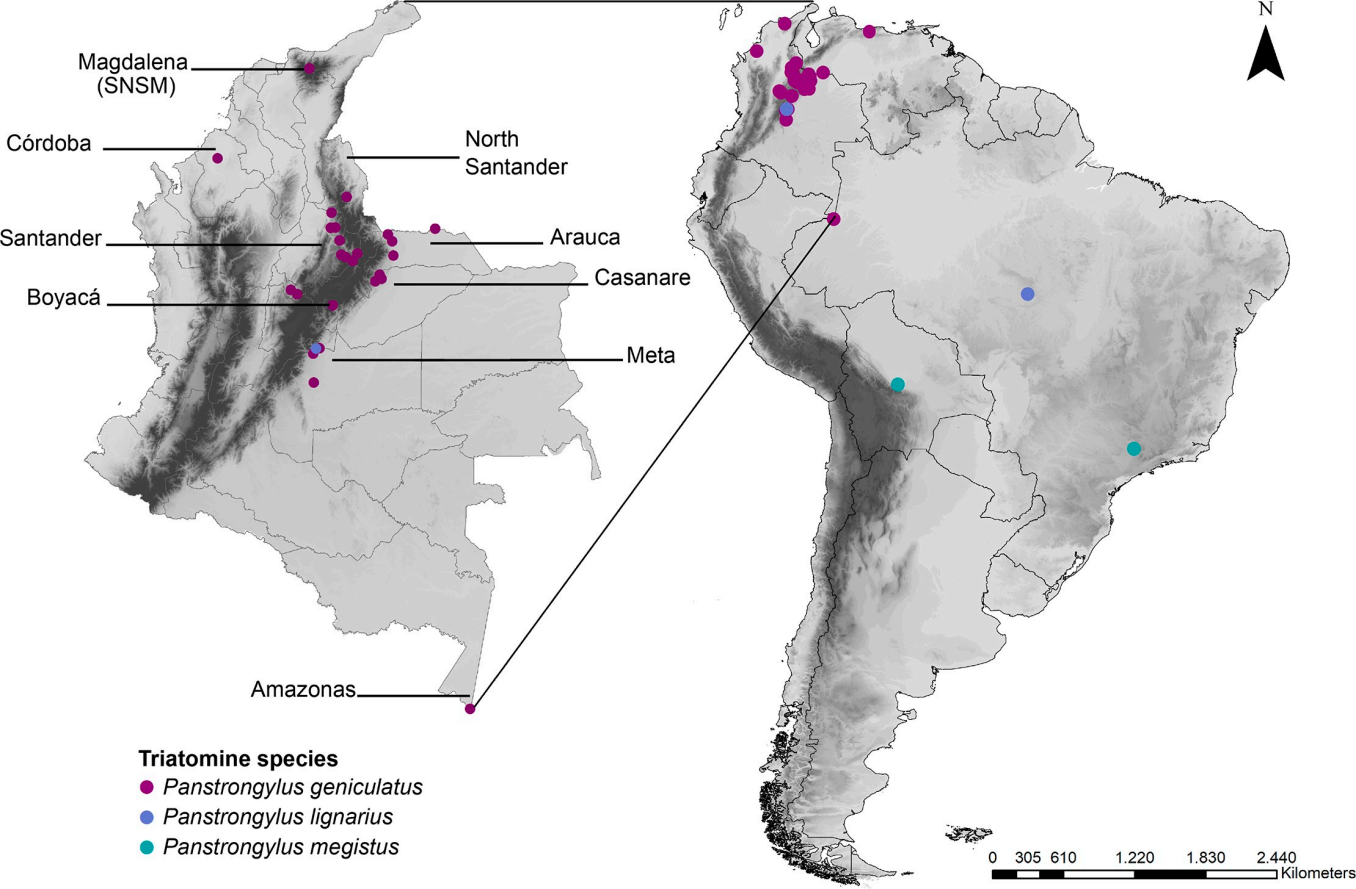
Map showing the locations of the samples collected in this study. The size of the circle is not representative of the number of individuals collected at each location. The map was constructed using QGIS version 2.18.7.

A total of 124 samples corresponding to three species (105 *P*. *geniculatus*, five *P*. *lignarius*, 14 *P*. *megistus*) were collected for this study. *Panstrongylus geniculatus* individuals were collected in Colombia and Venezuela (S1 Table; Fig 1). The remaining samples (*P*. *lignarius* and *P*. *megistus*) were collected in Brazil and Bolivia (S1 Table; Fig 1). Sylvatic ecotope individuals were collected using two techniques: manual search and modified Noireau baited chicken traps located in palm trees. The insects in the domestic ecotopes were collected by hand. All the specimens were identified with standard taxonomic keys [20], stored individually in plastic containers with 100% ethanol, and kept at −20°C until processing. UNIVERSIDAD DEL ROSARIO provided the field permit from ANLA (Autoridad Nacional de Licencias ambientales) 63257–2014. All collection was done on public land.

### DNA extraction, amplification, sequencing and alignment

DNA was extracted from the whole body (63 samples) and from the head, leg, and thorax (61 samples) using the DNeasy® Blood & Tissue kit (Qiagen, Germany) [21], but with double the amounts of buffer ATL, proteinase K, buffer AL, and ethanol. The final elution step was performed with only 150 μL of buffer AE. The concentration and quality of the DNA was measured as absorbance (A) ratios A_260_/A_280_ and A_230_/A_260_, respectively, with a NanoDrop 1000 spectrophotometer (Thermo Fisher Scientific Inc., MA, USA). The quality of the DNA was also verified on 2% agarose gels.

We used two sets of primers to amplify the nuclear ribosomal RNA (rRNA) genes, *18S* rRNA (823 bp) [19] and *28S* rRNA (696 bp) [22], and three sets of primers to amplify mitochondrial gene fragments: 630 bp of the NADH dehydrogenase subunit 4 gene, *ND4* [23], 552 bp of the cytochrome b gene, *Cytb* [24,25], and 508 bp of *16S* rRNA [19] (See S2 Table). The PCRs for all gene fragments were conducted in a final volume of 12.5 μl containing 1.5 μl of DNA template, 6.25 μl of GoTaq Green Master Mix (2×), 1.25 μl of each primer (10 μM), and 2.25 μl of nuclease-free water. The thermal cycling conditions have been reported elsewhere (see references in S2 Table). For all loci, we visualized 2 μL of the PCR product on a 1.5% agarose gel stained with SYBR® Safe DNA Gel Stain to verify the success of the PCR. Samples that showed a solid band of the expected size were purified with ExoSAP-IT and sequenced with Sanger sequencing (both strands) by Macrogen (Seoul, South Korea) using an ABI Prism 3100 Genetic Analyzer. The output sequences were read and assembled into contigs with Geneious 11.0.5 [26]. After base calling and editing, we used the same program to obtain a consensus sequence for each sample. A Geneious plug-in was used to align each locus with Muscle [27]. The resulting alignments were manually inspected for misalignments and ambiguities (S3 Table). In cases where ambiguities of bases were present, the different haplotypes were inferred for each locus with the PHASE algorithm implemented in DnaSP v5.10 [28], with 1000 iterations per simulation.

### Phylogenetic analyses and species tree reconstruction

We only used the mitochondrial gene fragments for these analyses because the nuclear ribosomal loci did not resolve *P*. *geniculatus* intraspecifically (see results; S1 Fig). To determine the phylogenetic position of *P*. *geniculatus* within the genus *Panstrongylus*, we included additional mitochondrial gene fragment sequences (i.e., *16S* rRNA, *Cytb*) available in GenBank for the following eight species: *P*. *geniculatus*, *P*. *megistus*, *P*. *lignarius*, *P*. *lutzi*, *P*. *tupynambai*, *P*. *chinai*, *P*. *rufotuberculatus* and *P*. *howardi* (see S4 Table for accession numbers).

We estimated the phylogenetic relationships using two tree inference criteria: maximum likelihood (ML) and Bayesian inference (BI). For the ML tree, we used the concatenated alignment (individual analyses per gene fragment were also performed [see S2 Fig]) with an unlinked substitution model per locus in IQ-Tree [29]. The best-fit nucleotide substitution model for each gene fragment was obtained with IQ-tree model selection based on the Bayesian information criterion [30], and the node supports were calculated with 10,000 ultrafast bootstrap pseudoreplicates. Ultrafast bootstrap values > 95% were deemed trustworthy [31]. For *Cytb*, we included sequences from Venezuela previously used in [32].

The BI reconstruction was implemented in MrBayes v. 3.2.6 [33]. We performed one run with four Markov chains (MCMC) for 100 million generations, sampling every 1000 generations and discarding the first 10% as burn-in. We calculated the posterior probability (PP) of the nodes based on the 50% majority consensus of the best tree distribution. All tree distributions were checked for convergence using Tracer v. 1.7.1 [34]. Both the best ML and BI topologies were rooted to the mid-point because we were looking at the relationships within *Panstrongylus*. The trees were visualized and edited with FigTree v.1.4.4 [35].

The species tree and time of divergence estimates were based on the monophyletic clades from the phylogenetic reconstructions. We identified four possible independent lineages of *P*. *geniculatus* and the other six lineages represented the rest of the *Panstrongylus* species included in this study. We estimated a species tree with three partitions, one for each gene fragment, using a coalescence-based approach implemented in StarBEAST2 v 2.4 [36,37]. We performed three independent runs of 1 × 10^8^ generations, sampled every 1000 generations, and discarded the first 15% as the burn-in period. The models of substitutions (estimated in IQ-tree) and priors were specified as follow (otherwise as default): *Cytb*, TIM2 + F + I + G4; *16S* rRNA, TPM2 + F + G4; *ND4*, HKY + F + I + G4 (models); relaxed uncorrelated lognormal clock (estimate); Yule process of speciation; and gene tree ploidy were mitochondrial (priors). We calibrated the root node of the species tree using the crown fossil *P*. *hispaniolae* (PaleoDatabase 49461, early Miocene), and used a uniform prior distribution with the following upper and lower bound range (20.44–13.82 Ma) [38]. We assessed the convergence of the model by examining the trace files in Tracer v.1.7.1 [34], after obtaining an effective sample size of > 200 for all parameters. We combined the tree files (two runs) using LogCombiner v.1.10.4 [37], and the uncertainty of the trees was visualized with DensiTree v.2.1 [37]. However, the maximum credibility trees with the coalescence-based divergence times and 95% highest probability densities were produced in Tree Annotator v.1.10.4 [36]. The species tree and divergence times were visualized with FigTree v.1.4.4 [35].

### Population genetics

We calculated haplotype networks for each locus using the available *Panstrongylus* species to visualize the haplotype diversity and clustering. We estimated these networks with a minimum distance algorithm in the program PopArt [39]. The haplotypic structure within *P*. *geniculatus* was assessed with the same approach.

Genetic diversity statistics were calculated for the clusters determined with the haplotype networks: nucleotide diversity (π), substitution rate (θ), number of segregating sites (S), and Tajima’s D. These statistics were calculated with DNASP v5.10 [28]. The genetic structure between the species and geographic groups was estimated with F_ST_ [40]_,_ D_XY_, and Da [41]. The effect of isolation by distance on the population structure was evaluated with the Mantel test [42] using the R package *vegan* [43]. To do so, a matrix of genetic distances was estimated by linearizing the F_ST_ values [44], and the pairwise geographic distances among localities were processed with the function *distm* in the R package *geosphere* [45]. We also examined the linear correlation between the geographic distances and genetic distances as recommended by Legendre et al. [46], with the entire dataset, but without extreme points. Because we were mainly interested in the diversification of *P*. *geniculatus*, all the summary statistics and structure analyses were performed with the samples collected for this study, including *P*. *megistus* and *P*. *lignarius*.

## Results

### Phylogenetic analyses and species tree reconstruction

**Fig 2:**
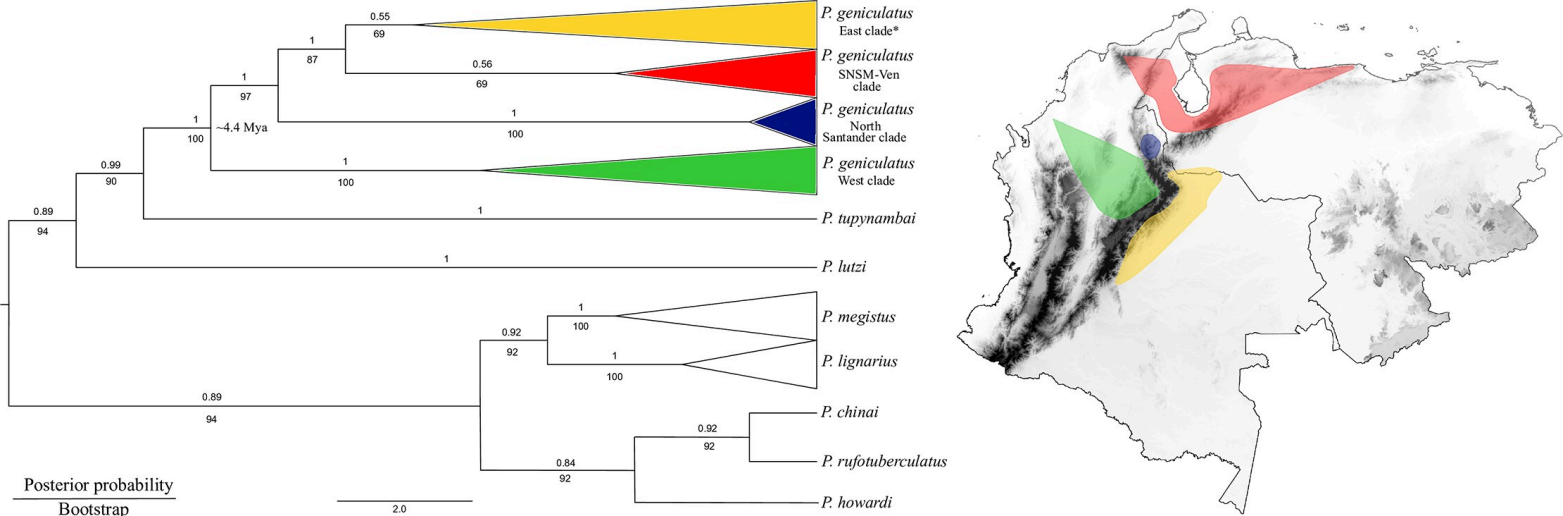
BI consensus phylogenetic tree inferred from mitochondrial DNA and distribution of the geographical clades. Numbers at the nodes are the bootstrap/posterior probability support values. Distributions were calculated with minimum convex polygon and are color coded based on the tree clades. The map was constructed using QGIS version 2.18.7.

**Fig 3:**
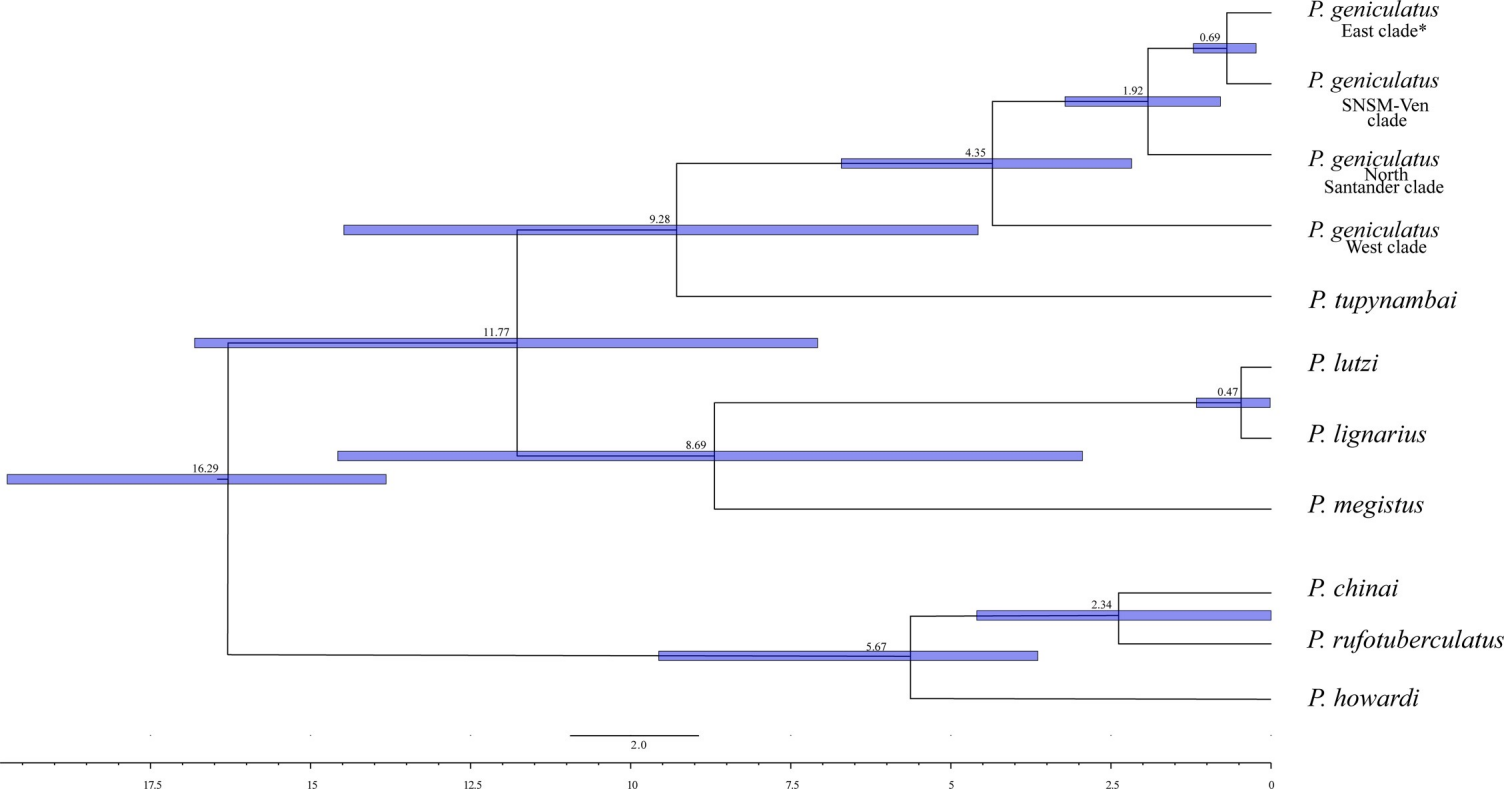
Dated coalescent Bayesian species tree estimated in *BEAST2. Numbers at the nodes are the estimated times of divergence. Each blue bar corresponds to the 95% highest posterior density (HDP) interval.

The ML and BI topologies for the mitochondrial gene fragments were completely concordant and revealed eight well-supported clades, corresponding to eight species (Fig 2). *Panstrongylus geniculatus* was monophyletic and had four clades (Fig 2), which were associated, to some extent, with its geographical range: 1) East of the Eastern Cordillera of the Colombian Andes clade (bootstrap support [BS] = 69, PP = 0.55), which included the departments Arauca, Casanare, Meta, Amazonas, and Córdoba, and some individuals from Santander, Boyacá, and Magdalena; 2) Magdalena (Sierra Nevada of Santa Marta, SNSM) with Venezuela clade (SNSM-Ven; BS = 69, PP = 0.56), which included all individuals from Venezuela and Magdalena; 3) North Santander clade (NSant; BS = 100, PP = 1), which only included the samples collected there; and 4) West of the Eastern Cordillera of the Colombian Andes clade (BS = 100, PP = 1), which included the rest of the Santander and Boyacá samples. However, the first two clades were not well-supported. *Panstrongylus geniculatus* diverged from *P*. *tupynambai* somewhere around 9.28 Mya (Confident Intervals, here after CI: 14.13–4.51 Mya, Fig 3), and the diversification within *P*. *geniculatus* occurred ~4.35 Mya (CI: 6.58–2.20 Mya, Fig 3). Finally, ML reconstruction for *Cytb* fragment including Venezuelan sequences obtained from [32] were recovered in the SNSM-Ven clade (S2 Fig) supporting the pattern previously described in the concatenated data.

### Population genetics

**Fig 4:**
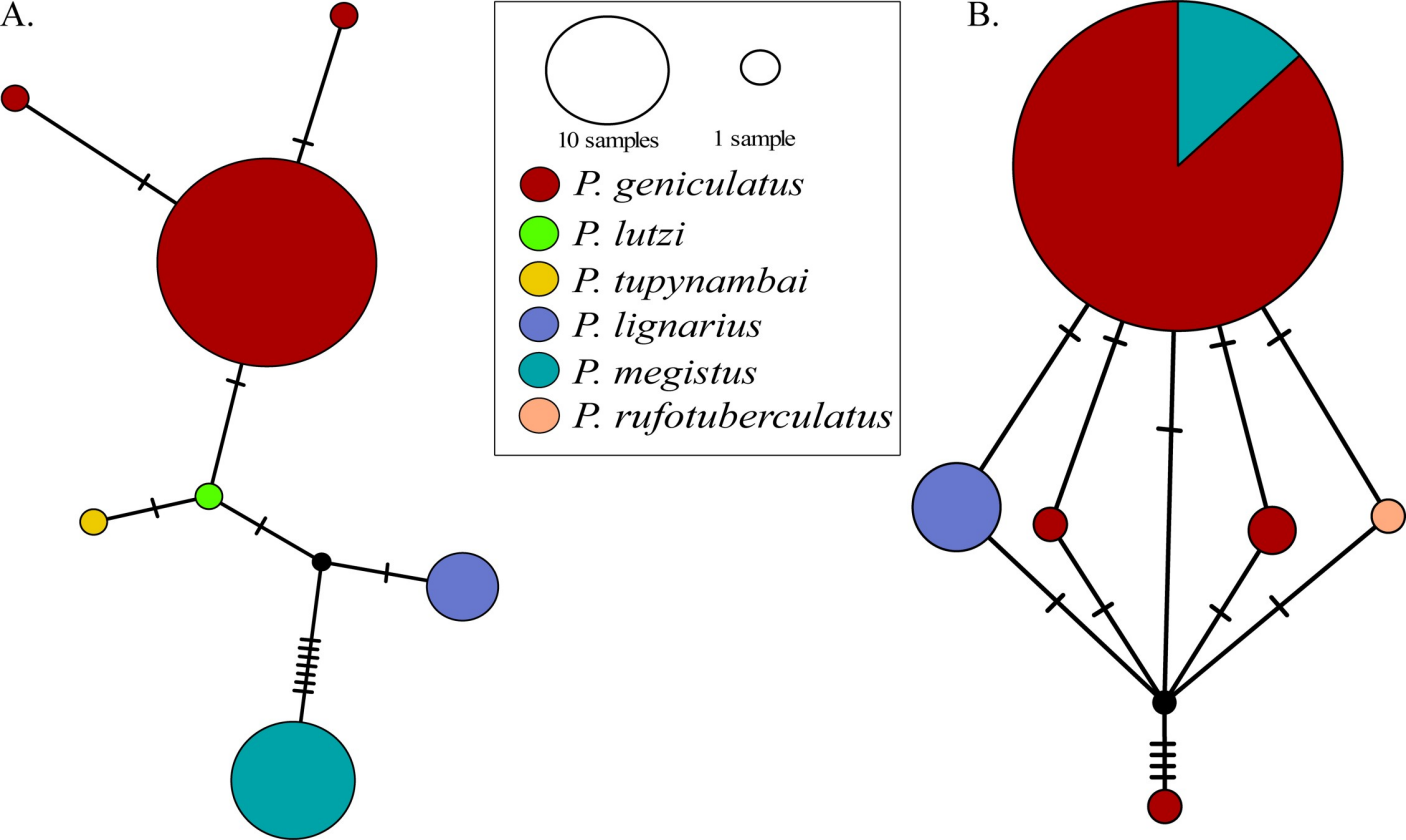
**Haplotype networks inferred with nuclear rRNA: A. *28S* and B. *18S*.** Each tick on the haplotype network represents a mutational step.

The two nuclear ribosomal loci showed low haplotype diversity (Fig 4). Specifically, the *28S* rRNA gene fragment showed that *P*. *geniculatus*, *P*. *megistus*, *P*. *lignarius*, *P*. *lutzi*, and *P*. *tupynambai* were separated clusters, although there were few mutational steps between each group (Fig 4), whereas the *18S* rRNA gene showed that *P*. *geniculatus* and *P*. *megistus* were intermixed.

**Fig 5:**
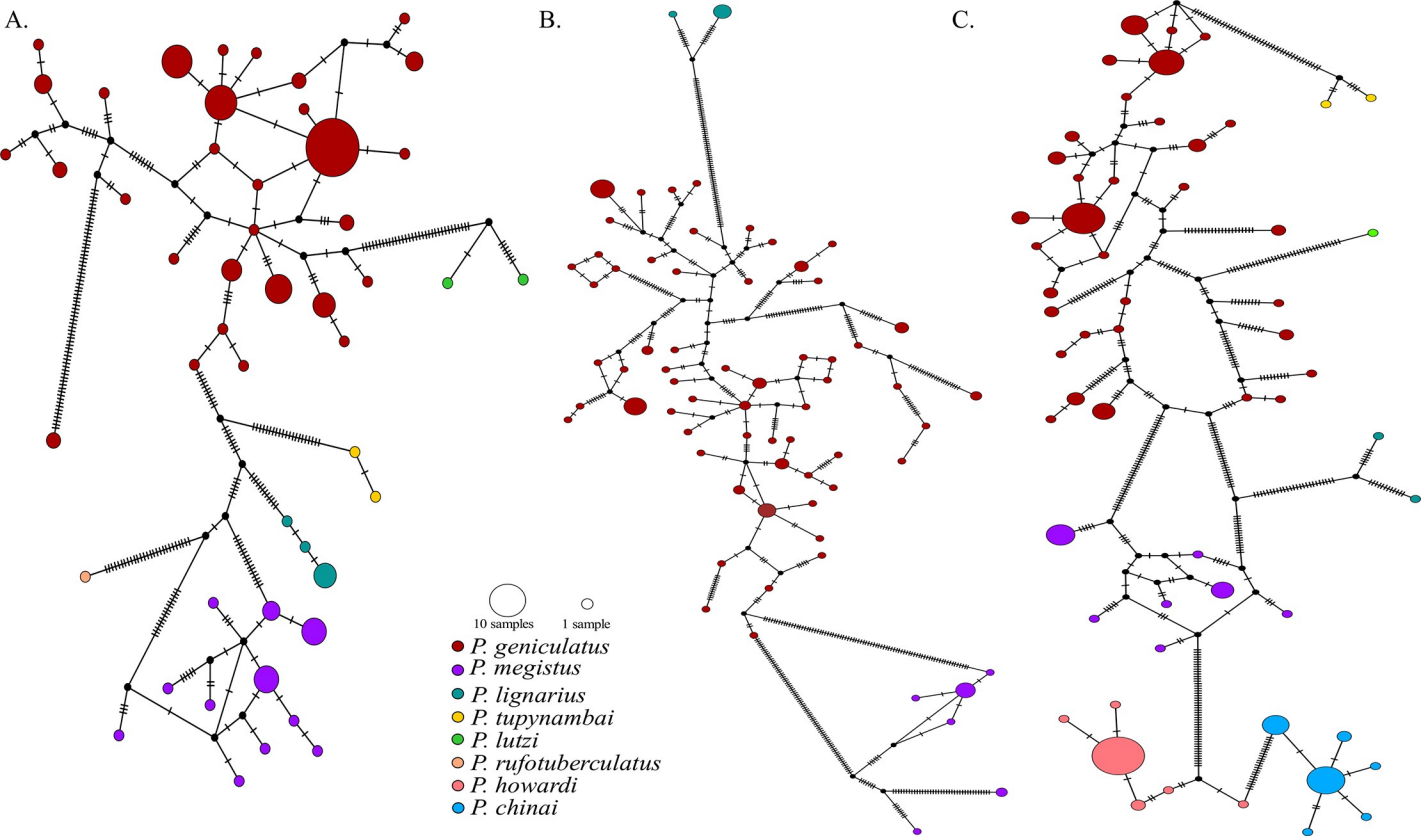
**Haplotype networks for mitochondrial loci grouped by species: A. *16S*, B. *ND4* and C. *Cytb*.** Each tick on the branches represents a mutational step.

**Fig 6:**
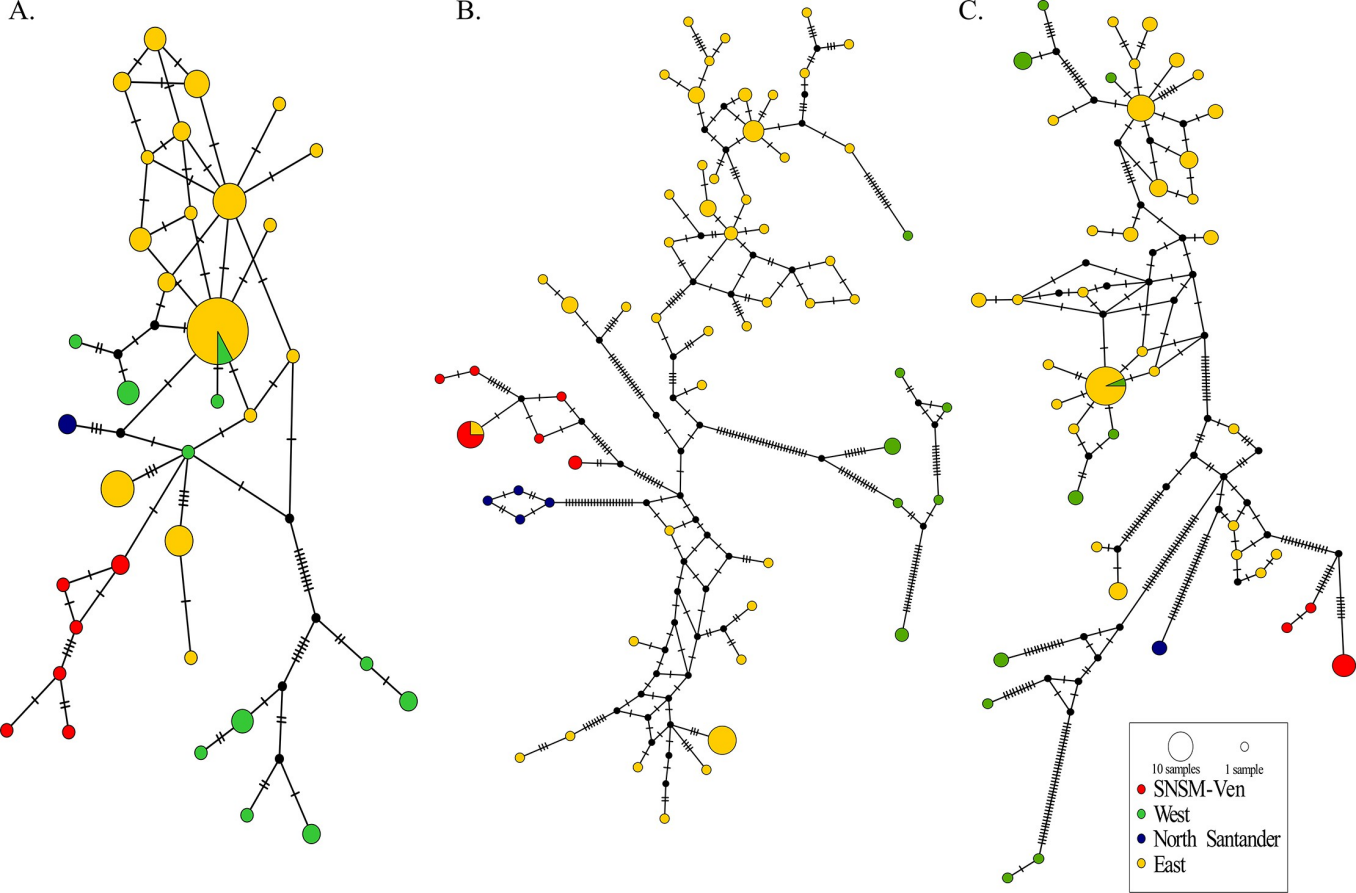
**Haplotype networks for mitochondrial loci grouped by geographic location: A. *16S*, B. *ND4* and C. *Cytb*.** Each tick on the branches represents a mutational step.

**Fig 7:**
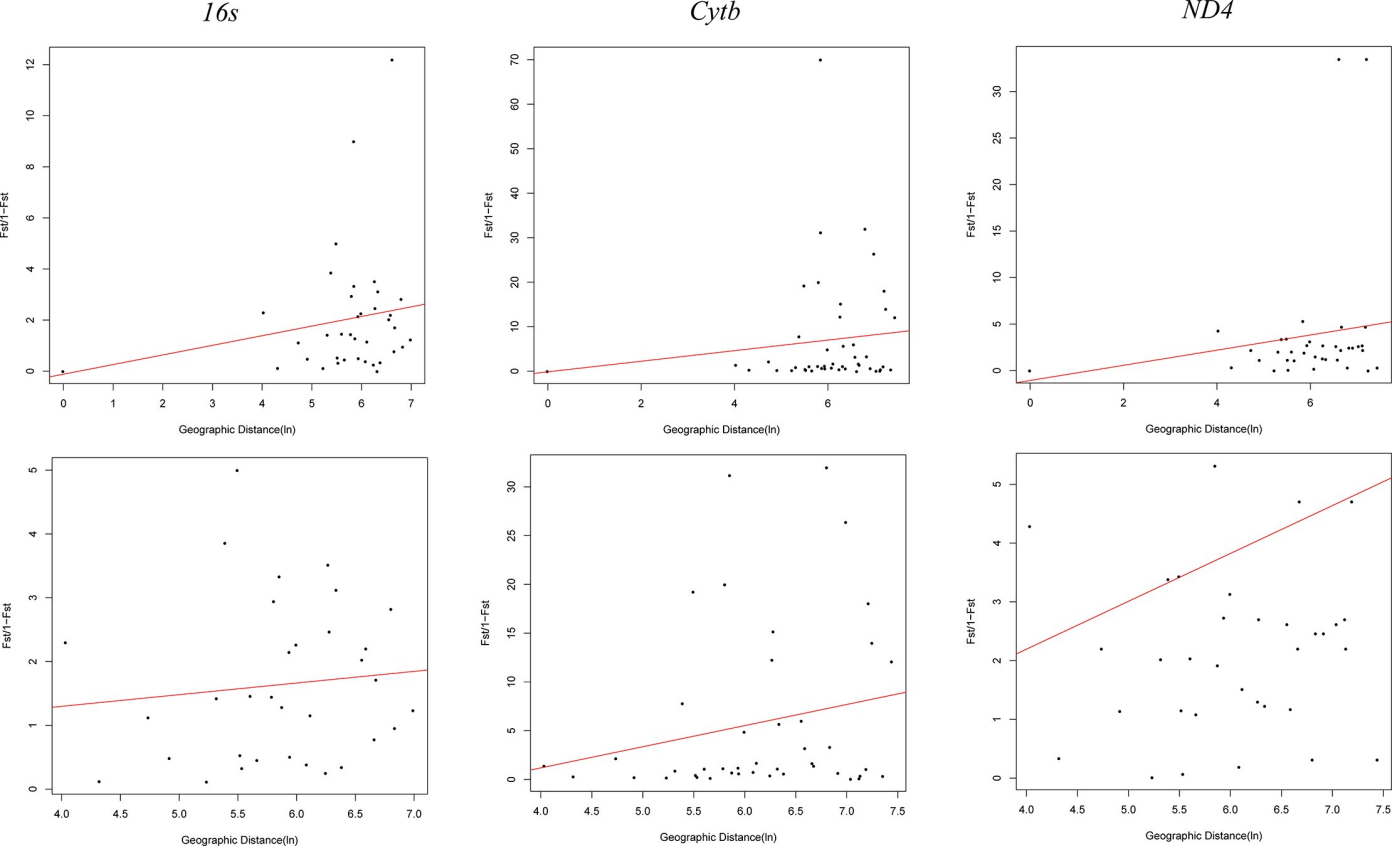
Isolation by distance plots for mitochondrial loci. Two tests of correlation were performed: with all the data (top) and without the extreme points (bottom).

The mitochondrial networks clustered the haplotypes by species, with many mutational steps among them (Fig 5). Consistent with this, we detected high genetic differentiation between the species (Table 1). However, nucleotide diversity was higher in *P*. *geniculatus* than in the other species (Table 2). A close examination of the *P*. *geniculatus* haplotype networks (Fig 6) confirmed that the previously described geographic groups (Fig 2) were genetically differentiated (Table 1). Isolation by distance was ruled out as a causal factor contributing to the geographic genetic structure (Table 3, Fig 7), and even though we found significant correlation between the geographic and genetic distances in some cases, the coefficient of determination of the linear model was poor and the Mantel test result was not significant (Table 3, Fig 7). We also found shared haplotypes in samples collected from both sides of the Eastern Cordillera (Fig 6 and S3 Fig). Specifically, these shared haplotypes involved individuals from the western localities of Santander, Boyacá, and Cordoba, which grouped with the samples from the east of the Eastern Cordillera. Our data also showed that the population in northwestern Colombia (SNSM) contained mitochondrial DNA (mtDNA) from east of the Eastern Cordillera.

**Table 1:** Measures of population structure between species and geographic clusters of *P*. *geniculatus* at mtDNA loci.

	Population1	*P*. *geniculatus*	*P*. *geniculatus*	*P*. *megistus*	SNSM-Ven	SNSM-Ven	SNSM-Ven	West	West	NSant
	Population2	*P*. *megistus*	*P*. *lignarius*	*P*. *lignarius*	West	NSant	East	NSant	East	East
***Cytb***	**F**_**ST**_	0.932	----	----	0.459	0.863	0.676	0.599	0.157	0.853
	**D**_**XY**_	0.161	----	----	0.084	0.082	0.068	0.082	0.052	0.075
	**Da**	0.123	----	----	0.039	0.071	0.047	0.049	0.008	0.064
	**P value**	0.00001***	----	----	0.00001***	0.039*	0.00001***	0.028*	0.00001***	0.001**
***ND4***	**F**_**ST**_	0.756	0.823	0.863	0.641	0.874	0.550	0.701	0.538	0.744
	**D**_**XY**_	0.158	0.159	0.155	0.085	0.061	0.045	0.093	0.088	0.062
	**Da**	0.119	0.131	0.134	0.057	0.053	0.025	0.065	0.047	0.046
	**P value**	0.00001***	0.00001***	0.00001***	0.00001***	0.002**	0.00001***	0.006**	0.00001***	0.00001***
***16S* rRNA**	**F**_**ST**_	0.911	0.910	0.969	0.417	0.756	0.456	0.539	0.328	0.713
	**D**_**XY**_	0.084	0.095	0.075	0.029	0.015	0.013	0.025	0.025	0.011
	**Da**	0.077	0.086	0.073	0.012	0.011	0.006	0.008	0.008	0.008
	**P value**	0.00001***	0.00001***	0.00001***	0.00001***	0.037*	0.00001***	0.005**	0.00001***	0.00001***
**Concatenated Data**	**F**_**ST**_	0.768	0.845	0.861	0.671	0.882	0.642	0.598	0.523	0.744
	**D**_**XY**_	0.143	0.125	0.142	0.074	0.039	0.041	0.063	0.049	0.053
	**Da**	0.112	0.104	0.102	0.033	0.039	0.022	0.047	0.023	0.041
	**P value**	0.00001***	0.00001***	0.00001***	0.00001***	0.0027**	0.00001***	0.002**	0.00001***	0.00001***

Probability calculated with the Hudson permutation test, with 1000 replicates.

*0.01 < P < 0.05

**0.001 < P < 0.01

***P < 0.001. “----”: some statistics could not be calculated due to low number of haplotypes per population.

**Table 2:** Genetic summary statistics for the three species analyzed in this study and for the Eastern, Western, North Santander (NSant) and Magdalena-Venezuela (SNSM-Ven) groups at each mitochondrial locus.

Genomic marker	Species/lineage	n	h	S	π ± SD	D
***Cytb***	***P*. *geniculatus***	134	61	136	0.051±0.002	−0.233
	***P*. *megistus***	13	2	21	0.020±0.003	2.539
	***P*. *lignarius***	1	1	0	0	----------
	**East**	61	30	54	0.022±0.002	0.185
	**NSant**	2	1	0	0	---------
	**SNSM-Ven**	57	25	70	0.022±0.003	-1.099
	**West**	14	10	99	0.066±0.010	0.734
***ND4***	***P*. *geniculatus***	92	60	145	0.045±0.003	−0.26792
	***P*. *megistus***	12	7	62	0.031±0.012	−0.28969
	***P*. *lignarius***	6	2	20	0.012±0.007	−1.49247
	**East**	66	44	76	0.028±0.002	0.081
	**NSant**	4	4	3	0.003±0.001	2.012
	**SNSM-Ven**	12	6	23	0.013±0.003	−0.050
	**West**	10	7	92	0.054±0.011	0.039
***16S* rRNA**	***P*. *geniculatus***	92	35	48	0.014±0.002	−0.84369
	***P*. *megistus***	14	4	4	0.003±0.001	1.05159
	***P*. *lignarius***	6	2	2	0.001±0.001	−1.13197
	**East**	65	18	16	0.006±0.001	-0.185
	**NSant**	2	1	0	0	---------
	**SNSM-Ven**	7	6	1	0.007±0.001	1.381
	**West**	18	11	34	0.027±0.002	1.400
**Concatenated Data**	**East**	70	61	497	0.005±0.003	-0.677
	**NSant**	4	4	3	0.002±0.001	2.011
	**SNSM-Ven**	12	7	11	0.016±0.01	1.032
	**West**	18	11	34	0.027±0.002	1.400

n: number of sequences; h: number of haplotypes; S: number of segregating sites; π: nucleotide diversity; D: Tajima’s D. None of the loci showed Tajima’s D values that departed from neutral expectations.

**Table 3:** Isolation by distance analysis. Mantel and correlation tests were performed with all the samples and without the extreme points.

	Entire Data					Excluding extreme points		
Locus	R	R^2^	P value	Mantel r	P value	R	R^2^	P value
***16S* rRNA**	0.3121	0.0974	0.0046**	0.1442	0.2548	0.8107	0.6572	0.5143
***Cytb***	0.1935	0.0374	0.0538	0.0672	0.3373	0.2025	0.041	0.0616
***ND4***	0.2422	0.0587	0.0294*	0.0998	0.2328	0.0813	0.0066	0.5164

P value:

*0.01 < P < 0.05

**0.001 < P < 0.01

## Discussion

Increasing evidence suggests that some species of *Panstrongylus* should be considered primary vectors of Chagas disease [1,5]. Despite this, few studies have reported the genetic diversity of these species. *Panstrongylus geniculatus* has become relevant over the years for three main reasons: its geographic distribution, its record of domiciliation, and its association with oral outbreaks of Chagas disease, mainly in Colombia and Venezuela. The phenotypic variability in this species has been studied at the morphological level [14–17], and only one karyotype study of *P*. *geniculatus* is known [47]. The observed intraspecific karyotypic and morphological diversity coupled to the wide distribution of *P*. *geniculatus* prompted these authors to propose that it is a complex of species [47]. However, this proposition remained to be tested at the molecular level.

### Phylogenetic relationships among *Panstrongylus* species

The phylogenetic relationships among *Panstrongylus* species are controversial. Few representatives of this genus were included in studies, which primary focus was to resolve the phylogenetic relationships within the subfamily Triatominae [19,48–51]. In these analyses, *Panstrongylus* was recovered as polyphyletic with *Triatoma* species [18–19,48,50–51]. Although, the aim of our study was to establish the intraspecific variation of *P*. *geniculatus*, we were able reconstruct the relationships of the *Panstrongylus* species included here from mtDNA analyses (Fig 2 and Fig 5). Two main clades were detected: the first one included *P*. *lutzi* and *P*. *tupynambai*, and this clade was sister to *P*. *geniculatus* (*geniculatus* group [48]). The second one contained *P*. *lignarius* and *P*. *megistus*, and this clade was sister to *P*. *chinai*, *P*. *rufotuberculatus* and *P*. *howardi*, as previously reported (*megistus* group [48]). This result contrasts with the previous lack of resolution obtained in other studies [18,49–51].

### Genetic variation and phylogeography of *P*. *geniculatus*

Our estimates of intraspecific genetic diversity were higher than the previously reported in two Caracas municipalities within Venezuela [32], due to a broader geographical sampling included in this study (Table 2). Genetic differences estimated between our geographical clades were higher than the values observed between populations occurring in a continuous space in Venezuela (without barriers) [32]. This could suggest that geographical barriers (such as the Andes) might promote stronger divergence than other eco-epidemiological factors. However, the measure of absolute divergence among the clades obtained in this study (D_XY_) was too low, suggesting that *P*. *geniculatus* is not a complex of species (Tables 1 and 2). In contrast, our nuclear rRNA dataset did not show the genetic structure detected with *P*. *geniculatus* mtDNA (Fig 4, S1 Fig). This can be explained by the differences in the coalescence times and effective population sizes in the nuclear rRNA and mtDNA analyses [52–54]. Our results based on mtDNA data suggest that the Andean orogeny might contributed to the structured geographic populations of *P*. *geniculatus*, as has been observed for other members of the Triatominae [48,55–57]. We found that the West and East clades of *P*. *geniculatus* diverged (Fig 3; 4.35 Mya [CI: 6.58–2.20 Mya]) within the period of the Andean uplift of the Colombian Eastern Cordillera, which achieved its final elevation ~2.5 Mya, during the Pliocene [58].

The rapid uplift of the Andes created a wide range of new ecological niches, with opportunities for colonization [58]. Interestingly, the shared haplotypes, with no evidence of isolation by distance, can be attributed to widely dispersed gene flow (Table 3, Fig 6, Fig 7, and S3 Fig). Thus, long-distance dispersal coupled to niche colonization could facilitate the admixtures of *P*. *geniculatus* populations that occur on opposite sides of the Andes. Interestingly, at the border between the Colombia East Andes and Mérida Andes in Venezuela, there are altitudinal depressions (e.g. Yaracuy and Táchira passes) that could facilitate genetic exchange among the *P*. *geniculatus* populations that occur on both sides of the Andes [59,60]. Geographic barriers, in this case the Andes uplift, have driven the diversification of many animals, including insects such as butterflies [61–63], bees [64], other triatomines [22,65–67], and arachnid species [68], among others. Moreover, gene flow by organismal dispersal through corridors has been documented in other arthropods [68–70].

### Vector importance of the *P*. *geniculatus* variability

Many studies have sought to explain the high morphological variability of certain species within the subfamily Triatominae. One of the most studied species is *Triatoma dimidiata* because of the high variance in its phenotypic and genetic data [23,71–72]. An investigation conducted in Colombia revealed a similar genetic structure [73] to the one found in this study. That structure correlated with eco-epidemiological and morphological traits, but not with geographic events that would favor the divergence of populations. Contrary to the lack of geographic association in that study, another research into different populations has suggested a major role for geological variation in the divergence within *T*. *dimidiata* [65]. This reflects the different ways in which the divergence of a population can occur. Geographic variation may explain its diversification, but so can orogenic and eco-epidemiological changes, among other factors [65–66,73]. *Panstrongylus geniculatus* could be experiencing a similar situation, insofar as geographic variation is not the only factor modeling the population structure. Mountains can also promote diversification through processes such as niche partitioning, altitudinal gradients, climatic variations, and long-distance dispersal, among other factors [22,65–67]. Other eco-epidemiological factors involving vector adaptability may also be shaping the divergence between *P*. *geniculatus* populations [65,74]. Further studies are required, with more markers or even genomes, and with more broadly sampled triatomine specimens, especially from other countries, to test the contributions of these factors to the diversification of this species.

The pattern of shared genetic variation between *P*. *geniculatus* geographical clades described before, could be explained using passive dispersion (e.g. vertebrate hosts) [75], considering that the adults of these species fly poorly [9, 72]. *Panstrongylus geniculatus* is commonly associated with opossums, armadillos, and bats [76], but more recent studies have demonstrated that this species has a wide range of feeding sources, including rodents, canines, ruminants and primates [1]. Its passive dispersal by human activity and with its carriage on animals is important for its long-distance migration, as previously described [74].

The broad spectrum of its ecological niches, coupled to its many feeding sources and its high level of genetic diversity demonstrated here (Table 1 and 2), increases the possibility of niche specialization in *P*. *geniculatus* and therefore the divergence of its populations. This is relevant because changes in the interaction between the vector and the host affect the dynamics of a disease [77]. If *P*. *geniculatus* is undergoing diversification, there will be new epidemiological challenges in the effective control of Chagas disease. The dispersal of the species is also highly relevant because of the incursion of sylvatic *T*. *cruzi* strains, which have been reported to be more virulent than domestic strains [7,8]. Mixed infections with the TcI, TcIII, and TcIV strains of *T*. *cruzi*, which are associated with sylvatic foci, have been reported [9], and there have also been reports of infection with the rest of the discrete typing units (DTUs) [1].

The success of vector control programs requires the accurate identification and incrimination of suspected vector species [78] and an understanding of the factors affecting transmission. These factors include the biological diversity, population dynamics, and spatial distribution of vector populations [77]. Specifically, the divergence of a species with a population structure can modify disease transmission and therefore alter the disease dynamics [77]. *Panstrongylus geniculatus* forms several vector populations, which could exploit several host populations. This research was a preliminary study to clarify the biology of *P*. *geniculatus* and may allow the development of effective strategies for its control, which should vary with the vector implicated [77].

## Conclusion

Our study represents the first multilocus approach to estimate the intraspecific relationships of *P*. *geniculatus* from northern South America. We identified four genetically differentiated clades, which could be associated with geographic events that shaped the demography of the species, including the uplift of the Eastern Cordillera of the Colombian Andes. Further studies with samples drawn from more widely spread locations, which consider vector adaptability factors, are required to extend our knowledge of this pathogen–vector interaction. This information should then be considered in the design of vector control strategies aimed to reduce the prevalence of Chagas disease.

## Supporting information

S1 Table Sampling used in this study. Last two letters of the ID correspond to species: Pg: *Panstrongylus geniculatus*, Pl: *P*. *lignarius* and Pm: *P*. *megistus*. Accession numbers: MK829849—MK 829943 (*Cytb*), MK829944—MK830032 (*ND4*), MK612546—MK6126555 (*16S*), MK612656—MK612759 (*18S*) and MK632224—MK632304 (*28S*).
(DOCX)

S2 Table Primers for nuclear ribosomal and mitochondrial gene fragments. (DOCX)

S3 Table Genetic marker, ID, and number of nucleotide ambiguities per sequence of *P*. *geniculatus*. (DOCX)

S4 Table Accession number of sequences downloaded from GenBank. (DOCX)

S1 Fig Phylogenetic trees inferred with nuclear ribosomal DNA for the loci evaluated. Numbers at the nodes are the bootstrap support values after 10,000 bootstrap replicates.
(TIF)

S2 Fig Phylogenetic trees inferred with mitochondrial DNA for the loci evaluated. Numbers at the nodes are the bootstrap support values after 10,000 bootstrap replicates.
(TIFF)

S3 Fig Consensus phylogenetic tree inferred from mitochondrial DNA. Numbers on the nodes are support values (bootstrap for maximum likelihood and posterior probability for Bayesian inference). Colors of individuals indicate their geographic origin (i.e., they belong to the clade of the same color). Tip labels correspond to the identifiers (IDs) described in S1 Table.
(TIFF)
