# Supplementary material for: Genetic diversification of Panstrongylus geniculatus (Reduviidae: Triatominae) in northern South America
Source: PLoS One. 2019 Oct 17;14(10):e0223963. doi: 10.1371/journal.pone.0223963 (PMC6797096; doi:10.1371/journal.pone.0223963)
Supplement: S1 Table — Last two letters of the ID correspond to species: Pg: Panstrongylus geniculatus, Pl: P. lignarius and Pm: P. megistus. Accession numbers: MK829849—MK 829943 (Cytb), MK829944—MK830032 (ND4), MK612546—MK6126555 (16S), MK612656—MK612759 (18S) and MK632224—MK632304 (28S). (DOCX) [file pone.0223963.s001.docx]

S1 Table. Sampling used in this study. Last two letters of the ID correspond to species: Pg: *Panstrongylus geniculatus*, Pl: *P. lignarius* and Pm: *P. megistus*. Accession numbers: MK829849 - MK 829943(*Cytb*), MK829944 - MK830032 (*ND4*), MK612546 - MK6126555 (*16S*), MK612656 - MK612759 (*18S*) and MK632224 - MK632304 (*28S*).

| **ID** | **Country** | **Department** | **Locality** | **Longitude** | **Latitude** |
| --- | --- | --- | --- | --- | --- |
| 1_NsantPg | Colombia | North Santander | Los patios (Santa clara) | -72.8464 | 7.8381 |
| 2_NsantPg | Colombia | North Santander | Los patios (Santa clara) | -72.8464 | 7.8381 |
| 3_AraucaPg | Colombia | Arauca | Saravena | -71.8767 | 6.9547 |
| 4_AraucaPg | Colombia | Arauca | Saravena | -71.8767 | 6.9547 |
| 5_AraucaPg | Colombia | Arauca | Saravena | -71.8767 | 6.9547 |
| 6_AraucaPg | Colombia | Arauca | Saravena | -71.8767 | 6.9547 |
| 7_AraucaPg | Colombia | Arauca | Tame | -71.7447 | 6.4583 |
| 9_SantPg | Colombia | Santander | Mogotes | -72.9703 | 6.4764 |
| 10_SantPg | Colombia | Santander | Macaravita | -72.5928 | 6.5067 |
| 11_AraucaPg | Colombia | Arauca | Arauca | -70.7617 | 7.0903 |
| 12_SantPg | Colombia | Santander | Lebrija | -73.2189 | 7.1131 |
| 13_SantPg | Colombia | Santander | Bucaramanga | -73.1161 | 7.1186 |
| 14_SantPg | Colombia | Santander | El Playón | -73.2033 | 7.4703 |
| 15_SantPg | Colombia | Santander | San Joaquín | -72.8675 | 6.4278 |
| 16_MetaPg | Colombia | Meta | Restrepo | -73.5642 | 4.2617 |
| 17_MetaPg | Colombia | Meta | Restrepo | -73.5642 | 4.2617 |
| 18_MetaPg | Colombia | Meta | Restrepo | -73.5642 | 4.2617 |
| 19_MetaPg | Colombia | Meta | Restrepo | -73.5642 | 4.2617 |
| 20_MetaPg | Colombia | Meta | Restrepo | -73.5642 | 4.2617 |
| 21_MetaPg | Colombia | Meta | Restrepo | -73.5642 | 4.2617 |
| 22_MetaPg | Colombia | Meta | Restrepo | -73.5642 | 4.2617 |
| 23_MetaPg | Colombia | Meta | Restrepo | -73.5642 | 4.2617 |
| 24_MetaPg | Colombia | Meta | Restrepo | -73.5642 | 4.2617 |
| 25_MetaPg | Colombia | Meta | Restrepo | -73.5642 | 4.2617 |
| 26_MetaPg | Colombia | Meta | Restrepo | -73.5642 | 4.2617 |
| 27_MetaPg | Colombia | Meta | Restrepo | -73.5642 | 4.2617 |
| 28_MetaPg | Colombia | Meta | Restrepo | -73.5642 | 4.2617 |
| 29_MetaPg | Colombia | Meta | Restrepo | -73.5642 | 4.2617 |
| 30_MetaPg | Colombia | Meta | Restrepo | -73.5642 | 4.2617 |
| 31_MetaPg | Colombia | Meta | Restrepo | -73.5642 | 4.2617 |
| 32_MetaPl | Colombia | Meta | Restrepo | -73.5642 | 4.2617 |
| 33_MetaPg | Colombia | Meta | Restrepo | -73.5642 | 4.2617 |
| 34_MetaPg | Colombia | Meta | Restrepo | -73.5642 | 4.2617 |
| 35_MetaPg | Colombia | Meta | Restrepo | -73.5642 | 4.2617 |
| 36_MetaPg | Colombia | Meta | Restrepo | -73.5642 | 4.2617 |
| 37_MetaPg | Colombia | Meta | Fuente de Oro | -73.6192 | 3.4625 |
| 38_MetaPg | Colombia | Meta | Cumaral | -73.4864 | 4.2694 |
| 39_MetaPg | Colombia | Meta | Restrepo | -73.5642 | 4.2617 |
| 40_MetaPg | Colombia | Meta | Restrepo | -73.5642 | 4.2617 |
| 41_MetaPg | Colombia | Meta | Restrepo | -73.5642 | 4.2617 |
| 42_MetaPg | Colombia | Meta | Restrepo | -73.5642 | 4.2617 |
| 43_MetaPg | Colombia | Meta | Restrepo | -73.5642 | 4.2617 |
| 44_MetaPg | Colombia | Meta | Restrepo | -73.5642 | 4.2617 |
| 45_MetaPg | Colombia | Meta | Restrepo | -73.5642 | 4.2617 |
| 46_MetaPg | Colombia | Meta | Restrepo | -73.5642 | 4.2617 |
| 47_MetaPg | Colombia | Meta | Villavicencio | -73.6294 | 4.1425 |
| 48_MetaPg | Colombia | Meta | Restrepo | -73.5642 | 4.2617 |
| 49_MetaPg | Colombia | Meta | Restrepo | -73.5642 | 4.2617 |
| 50_CasanarePg | Colombia | Casanare | Paz de Ariporo | -72.0722 | 6.0056 |
| 51_CasanarePg | Colombia | Casanare | Paz de Ariporo | -72.0722 | 6.0056 |
| 52_CasanarePg | Colombia | Casanare | Paz de Ariporo | -72.0722 | 6.0056 |
| 54_CasanarePg | Colombia | Casanare | Paz de Ariporo | -72.0722 | 6.0056 |
| 55_CasanarePg | Colombia | Casanare | Paz de Ariporo | -72.0722 | 6.0056 |
| 56_CasanarePg | Colombia | Casanare | Paz de Ariporo | -72.0722 | 6.0056 |
| 57_CasanarePg | Colombia | Casanare | Paz de Ariporo | -72.0722 | 6.0056 |
| 58_CasanarePg | Colombia | Casanare | Paz de Ariporo | -72.0722 | 6.0056 |
| 59_CasanarePg | Colombia | Casanare | Tamara | -72.0225 | 5.9111 |
| 60_CasanarePg | Colombia | Casanare | Paz de Ariporo | -72.0722 | 6.0056 |
| 61_CasanarePg | Colombia | Casanare | Paz de Ariporo | -72.0722 | 6.0056 |
| 62_CasanarePg | Colombia | Casanare | Tamara | -72.1667 | 5.85 |
| 63_CasanarePg | Colombia | Casanare | Tamara | -72.1667 | 5.85 |
| 64_CasanarePg | Colombia | Casanare | Tamara | -72.1667 | 5.85 |
| 65_CasanarePg | Colombia | Casanare | Tamara | -72.1667 | 5.85 |
| 66_CasanarePg | Colombia | Casanare | Paz de Ariporo | -72.0722 | 6.0056 |
| 67_CasanarePg | Colombia | Casanare | Paz de Ariporo | -72.0722 | 6.0056 |
| 68_CasanarePg | Colombia | Casanare | Paz de Ariporo | -72.0722 | 6.0056 |
| 69_CasanarePg | Colombia | Casanare | Paz de Ariporo | -72.0722 | 6.0056 |
| 70_AraucaPg | Colombia | Arauca | Arauca | -70.7617 | 7.0903 |
| 71_AraucaPg | Colombia | Arauca | Fortul | -71.7714 | 6.7931 |
| 72_AraucaPg | Colombia | Arauca | Tame | -71.7447 | 6.4583 |
| 73_AraucaPg | Colombia | Arauca | Saravena | -71.8767 | 6.9547 |
| 74_AraucaPg | Colombia | Arauca | Fortul | -71.7714 | 6.7931 |
| 75_AraucaPg | Colombia | Arauca | Puerto Rondón | -71.1003 | 6.28 |
| 76_AraucaPg | Colombia | Arauca | Arauca | -70.7617 | 7.0903 |
| 77_AraucaPg | Colombia | Arauca | Tame | -71.7447 | 6.4583 |
| 78_AraucaPg | Colombia | Arauca | Saravena | -71.8767 | 6.9547 |
| 79_AraucaPg | Colombia | Arauca | Saravena | -71.8767 | 6.9547 |
| 80_SantPg | Colombia | Santander | El Playon | -73.2033 | 7.4703 |
| 82_CordobaPg | Colombia | Córdoba | Siete Palmas | -75.8833 | 8.75 |
| 83_CordobaPg | Colombia | Córdoba | Siete Palmas | -75.8833 | 8.75 |
| 84_CordobaPg | Colombia | Córdoba | Siete Palmas | -75.8833 | 8.75 |
| 85_VenezuelaPg | Venezuela | Aragua | Choroní | -67.6063 | 10.5069 |
| 86_VenezuelaPg | Venezuela | Aragua | Choroní | -67.6063 | 10.5069 |
| 87_VenezuelaPg | Venezuela | Aragua | Choroní | -67.6063 | 10.5069 |
| 88_VenezuelaPg | Venezuela | Aragua | Chroroní | -67.6063 | 10.5069 |
| 89_VenezuelaPg | Venezuela | Aragua | Choroní | -67.6063 | 10.5069 |
| 90_BrazilPm | Brazil | São Paulo | São João da Boa Vista | -46.7978 | -21.9689 |
| 91_BrazilPm | Brazil | São Paulo | São João da Boa Vista | -46.7978 | -21.9689 |
| 92_BrazilPm | Brazil | São Paulo | São João da Boa Vista | -46.7978 | -21.9689 |
| 93_BrazilPm | Brazil | São Paulo | São João da Boa Vista | -46.7978 | -21.9689 |
| 94_BrazilPm | Brazil | São Paulo | São João da Boa Vista | -46.7978 | -21.9689 |
| 95_BrazilPl | Brazil | Maranhão | São Raimundo das Mangabeiras | -45.4808 | -7.0219 |
| 96_BrazilPl | Brazil | Maranhão | São Raimundo das Mangabeiras | -45.4808 | -7.0219 |
| 97_BrazilPl | Brazil | Maranhão | São Raimundo das Mangabeiras | -45.4808 | -7.0219 |
| 98_BrazilPl | Brazil | Maranhão | São Raimundo das Mangabeiras | -45.4808 | -7.0219 |
| 99_BrazilPl | Brazil | Maranhão | São Raimundo das Mangabeiras | -45.4808 | -7.0219 |
| 100_BoyacaPg | Colombia | Boyacá | Zetáquira | -73.1733 | 5.28556 |
| 101_BoyacaPg | Colombia | Boyacá | Susacón | -73.01 | 6.816 |
| 102_BoyacaPg | Colombia | Boyacá | Susacón | -73.01 | 6.816 |
| 103_BoyacaPg | Colombia | Boyacá | Soata | -72.7 | 6.3333 |
| 104_BoyacaPg | Colombia | Boyacá | Soata | -72.7 | 6.3333 |
| 105_BoyacaPg | Colombia | Boyacá | San Pablo de Borbur | -74.155 | 5.6511 |
| 106_BoyacaPg | Colombia | Boyacá | Maripi | -74.0086 | 5.5519 |
| 107_BoyacaPg | Colombia | Boyacá | Maripi | -74.0086 | 5.5519 |
| 108_BoliviaPm | Bolivia | Cochamba | Aiquile | -65.1919 | -18.2605 |
| 109_BoliviaPm | Bolivia | Cochamba | Aiquile | -65.1919 | -18.2605 |
| 110_BoliviaPm | Bolivia | Cochamba | Aiquile | -65.1919 | -18.2605 |
| 111_BoliviaPm | Bolivia | Cochamba | Aiquile | -65.1919 | -18.2605 |
| 112_BoliviaPm | Bolivia | Cochamba | Aiquile | -65.1919 | -18.2605 |
| 113_BoliviaPm | Bolivia | Cochamba | Aiquile | -65.1919 | -18.2605 |
| 114_BoliviaPm | Bolivia | Cochamba | Aiquile | -65.1919 | -18.2605 |
| 115_BoliviaPm | Bolivia | Cochamba | Aiquile | -65.1919 | -18.2605 |
| 116_BoliviaPm | Bolivia | Cochamba | Aiquile | -65.1919 | -18.2605 |
| 117_SNSMPg | Colombia | Magdalena | Sierra Nevada of Santa Marta | -73.72 | 10.8667 |
| 118_SNSMPg | Colombia | Magdalena | Sierra Nevada of Santa Marta | -73.72 | 10.8667 |
| 119_SNSMPg | Colombia | Magdalena | Sierra Nevada of Santa Marta | -73.72 | 10.8667 |
| 120_SNSMPg | Colombia | Magdalena | Sierra Nevada of Santa Marta | -73.72 | 10.8667 |
| 121_SNSMPg | Colombia | Magdalena | Sierra Nevada of Santa Marta | -73.72 | 10.8667 |
| 122_SNSMPg | Colombia | Magdalena | Sierra Nevada of Santa Marta | -73.72 | 10.8667 |
| 127_LeticiaPg | Colombia | Amazonas | Leticia | -69.953 | -4.1275 |
| 128_LeticiaPg | Colombia | Amazonas | Leticia | -69.953 | -4.1275 |
| 129_LeticiaPg | Colombia | Amazonas | Leticia | -69.953 | -4.1275 |
| 130_LeticiaPg | Colombia | Amazonas | Leticia | -69.953 | -4.1275 |
| 131_LeticiaPg | Colombia | Amazonas | Leticia | -69.953 | -4.1275 |
