# Supplementary material for: Genetic diversification of Panstrongylus geniculatus (Reduviidae: Triatominae) in northern South America
Source: PLoS One. 2019 Oct 17;14(10):e0223963. doi: 10.1371/journal.pone.0223963 (PMC6797096; doi:10.1371/journal.pone.0223963)
Supplement: S2 Table — (DOCX) [file pone.0223963.s002.docx]

**S2 Table. Primers for nuclear ribosomal and mitochondrial gene fragments.**

| **Gene fragment** | **Primers** | **Region amplified (bp)** |
| --- | --- | --- |
| ͣ NADH dehydrogenase subunit 4, *ND4* [23] | F: TCAACATGAGCCCTTGGAAG | 630 |
|  | R: ATTCGTTGTCATGGTAATG |  |
| *ͣ* Cytochrome b, *Cytb* [24,25] | F: GGACG(AT)GG(AT)ATTTATTATGGATC | 522 |
|  | R: ATTACTCCTCCTAGYTTATTAGGAATT |  |
| ͣ16S [19] | F: CGCCTGTTTATCAAAAACAT | 508 |
|  | R: CTCCGGTTTGAACTCAGATCA |  |
| ᵇ18S [19] | F: AAATTACCCACTCCCGGCA | 823 |
|  | R: TGGTGUGGTTTCCCGTGTT |  |
| ᵇ28S [22] | F: GCGAGTCGTGTTGCTTGATAGTGCAG | 696 |
|  | R: TTGGTCCGTGTTTCAAGACGGG |  |

ͣMitochondrial marker; ᵇnuclear ribosomal (rRNA) markers. F: Forward Primer and R: Reverse Primer. References according to the main text.
