# Supplementary material for: Genetic diversification of Panstrongylus geniculatus (Reduviidae: Triatominae) in northern South America
Source: PLoS One. 2019 Oct 17;14(10):e0223963. doi: 10.1371/journal.pone.0223963 (PMC6797096; doi:10.1371/journal.pone.0223963)
Supplement: S3 Table — (DOCX) [file pone.0223963.s003.docx]

**S3 Table. Genetic marker, ID, and number of nucleotide ambiguities per sequence of *P. geniculatus***

| **Genomic marker** | **Sequence ID** | **W** | **M** | **Y** | **R** |
| --- | --- | --- | --- | --- | --- |
| **16S rRNA** | 26-MetaPg | - | 1/508 | - | - |
|  | 122-SNSMPg | 1/508 | - | - | - |
| ***Cytb*** | 31-MetaPg | - | - | 11/550 | 2/550 |
|  | 84-CordobaPg | - | - | 1/550 | 2/550 |
|  | 130-Leticiag | - | - | 1/550 | - |
| ***ND4*** | 23-MetaPg | - | - | 4/630 | 1/630 |
|  | 29-MetaPg | - | - | 1/630 | - |
|  | 39-MetaPg | - | - | 4/630 | 1/630 |
|  | 42-MetaPg | - | - | - | 1/630 |
|  | 45-MetaPg | - | - | 6/630 | 4/630 |
|  | 54-CasanarePg | - | - | 6/630 | 3/630 |
|  | 59-CasanarePg | - | - | 7/630 | 6/630 |
|  | 61-CasanarePg | - | - | 3/630 | 3/630 |
|  | 70-AraucaPg | - | 1/630 | - | - |
|  | 72-AraucaPg | - | - | 2/630 | 1/630 |
|  | 76-AraucaPg | - | - | - | 2/630 |
|  | 79-AraucaPg | - | - | 1/630 | 3/630 |
|  | 121-SNSMPg | - | - | 2/630 | - |

Nucleotide ambiguity codes based on IUPAC designations: W = A/T; M = A/C; Y = A/C, and R = A/G. Numbers in each cell represent the number of sites with an IUPAC code in the total length of the sequence for each gene.
