# Supplementary material for: Genetic diversification of Panstrongylus geniculatus (Reduviidae: Triatominae) in northern South America
Source: PLoS One. 2019 Oct 17;14(10):e0223963. doi: 10.1371/journal.pone.0223963 (PMC6797096; doi:10.1371/journal.pone.0223963)
Supplement: S4 Table — (DOCX) [file pone.0223963.s004.docx]

S2 Table. Accession numbers of sequences downloaded from GenBank for each gene.

| **Species** | ***18s*** | ***28s*** | ***16s*** | ***Cytb*** |
| --- | --- | --- | --- | --- |
| *P. lutzi* |  | KC249135.1 | KC248969.1 | KC249227.1 |
|  |  |  | AY035449.1 |  |
| *P. tupynambai* |  |  | KC248977.1 | KC249233.1 |
|  |  |  | KC248978.1 | KC249234.1 |
| *P. geniculatus* | JQ897583.1 | JQ897655.1 | AF394593.1 | KX109903.1 |
|  | JQ897585.1 | JQ897657.1 | JQ897822.1 | AF045722.1 |
|  |  | KX109907.1 | JQ897824.1 |  |
| *P. megistus* | AJ243336.1 | GQ853381.1 | AF021177.1 | JX088615.1 |
|  |  | KC249136.1 | AF021181.1 | JX088616.1 |
|  |  | KC249137.1 | AF028741.1 | JX088617.1 |
|  |  | KC249138.1 | EU827205.1 | JX088618.1 |
|  |  | KC249139.1 | EU827224.1 | JX088619.1 |
|  |  | KC249140.1 | EU827233.1 | JX088620.1 |
|  |  | KC249141.1 | EU827234.1 | JX088621.1 |
|  |  |  | EU835979.1 | JX088622.1 |
|  |  |  | KC248970.1 | JX088623.1 |
|  |  |  | KC248971.1 | JX088624.1 |
|  |  |  | KC248972.1 | JX088625.1 |
|  |  |  | KC248973.1 | JX088626.1 |
|  |  |  | KC248974.1 | JX088627.1 |
|  |  |  | KC248975.1 | KC249228.1 |
|  |  |  | KC248976.1 | KC249229.1 |
|  |  |  |  | KC249230.1 |
|  |  |  |  | KC249231.1 |
|  |  |  |  | KC249232.1 |
|  |  |  |  | AF045722.1 |
| *P. lignarius* | JQ897584.1 | JQ897656.1 | JQ897823.1 | KX109902.1 |
|  |  | KX109906.1 | KY748238.1 |  |
| *P. rufotuberculatus* |  |  | KY748239.1 | JX088625.1 |
|  |  |  |  | JX088626.1 |
|  |  |  |  | JX400931.1 |
|  |  |  |  | JX400932.1 |
|  |  |  |  | JX400963.1 |
|  |  |  |  | JX400976.1 |
|  |  |  |  | JX400978.1 |
|  |  |  |  | JX400989.1 |
|  |  |  |  | JX400990.1 |
|  |  |  |  | JX400991.1 |
|  |  |  |  | JX400991.1 |
|  |  |  |  | JX400992.1 |
|  |  |  |  | JX400993.1 |
|  |  |  |  | JX400995.1 |
| *P. chinai* |  |  |  | JX400933.1 |
|  |  |  |  | JX400934.1 |
|  |  |  |  | JX400935.1 |
|  |  |  |  | JX400942.1 |
|  |  |  |  | JX400943.1 |
|  |  |  |  | JX400944.1 |
|  |  |  |  | JX400945.1 |
|  |  |  |  | JX400954.1 |
|  |  |  |  | JX400955.1 |
|  |  |  |  | JX400956.1 |
|  |  |  |  | JX400957.1 |
|  |  |  |  | JX400958.1 |
|  |  |  |  | JX400959.1 |
|  |  |  |  | JX400960.1 |
|  |  |  |  | JX400961.1 |
|  |  |  |  | JX400962.1 |
|  |  |  |  | JX400964.1 |
|  |  |  |  | JX400965.1 |
|  |  |  |  | JX400966.1 |
|  |  |  |  | JX400967.1 |
|  |  |  |  | JX400971.1 |
|  |  |  |  | JX400974.1 |
|  |  |  |  | JX400975.1 |
|  |  |  |  | JX400979.1 |
|  |  |  |  | JX400982.1 |
|  |  |  |  | JX400983.1 |
| *P. howardi* |  |  |  | JX400936.1 |
|  |  |  |  | JX400937.1 |
|  |  |  |  | JX400938.1 |
|  |  |  |  | JX400939.1 |
|  |  |  |  | JX400940.1 |
|  |  |  |  | JX400941.1 |
|  |  |  |  | JX400946.1 |
|  |  |  |  | JX400947.1 |
|  |  |  |  | JX400948.1 |
|  |  |  |  | JX400949.1 |
|  |  |  |  | JX400950.1 |
|  |  |  |  | JX400951.1 |
|  |  |  |  | JX400952.1 |
|  |  |  |  | JX400953.1 |
|  |  |  |  | JX400968.1 |
|  |  |  |  | JX400969.1 |
|  |  |  |  | JX400970.1 |
|  |  |  |  | JX400972.1 |
|  |  |  |  | JX400973.1 |
|  |  |  |  | JX400980.1 |
|  |  |  |  | JX400981.1 |
|  |  |  |  | JX400984.1 |
|  |  |  |  | JX400985.1 |
|  |  |  |  | JX400986.1 |
|  |  |  |  | JX400987.1 |
|  |  |  |  | JX400988.1 |
|  |  |  |  | JX400994.1 |
|  |  |  |  | JX400996.1 |
|  |  |  |  | JX400997.1 |
|  |  |  |  | JX400998.1 |
|  |  |  |  | JX400999.1 |
|  |  |  |  | JX401000.1 |
|  |  |  |  | JX401001.1 |
