# Supplementary figures and images for: Genetic diversification of Panstrongylus geniculatus (Reduviidae: Triatominae) in northern South America
Source: PLoS One. 2019 Oct 17;14(10):e0223963. doi: 10.1371/journal.pone.0223963 (PMC6797096; doi:10.1371/journal.pone.0223963)

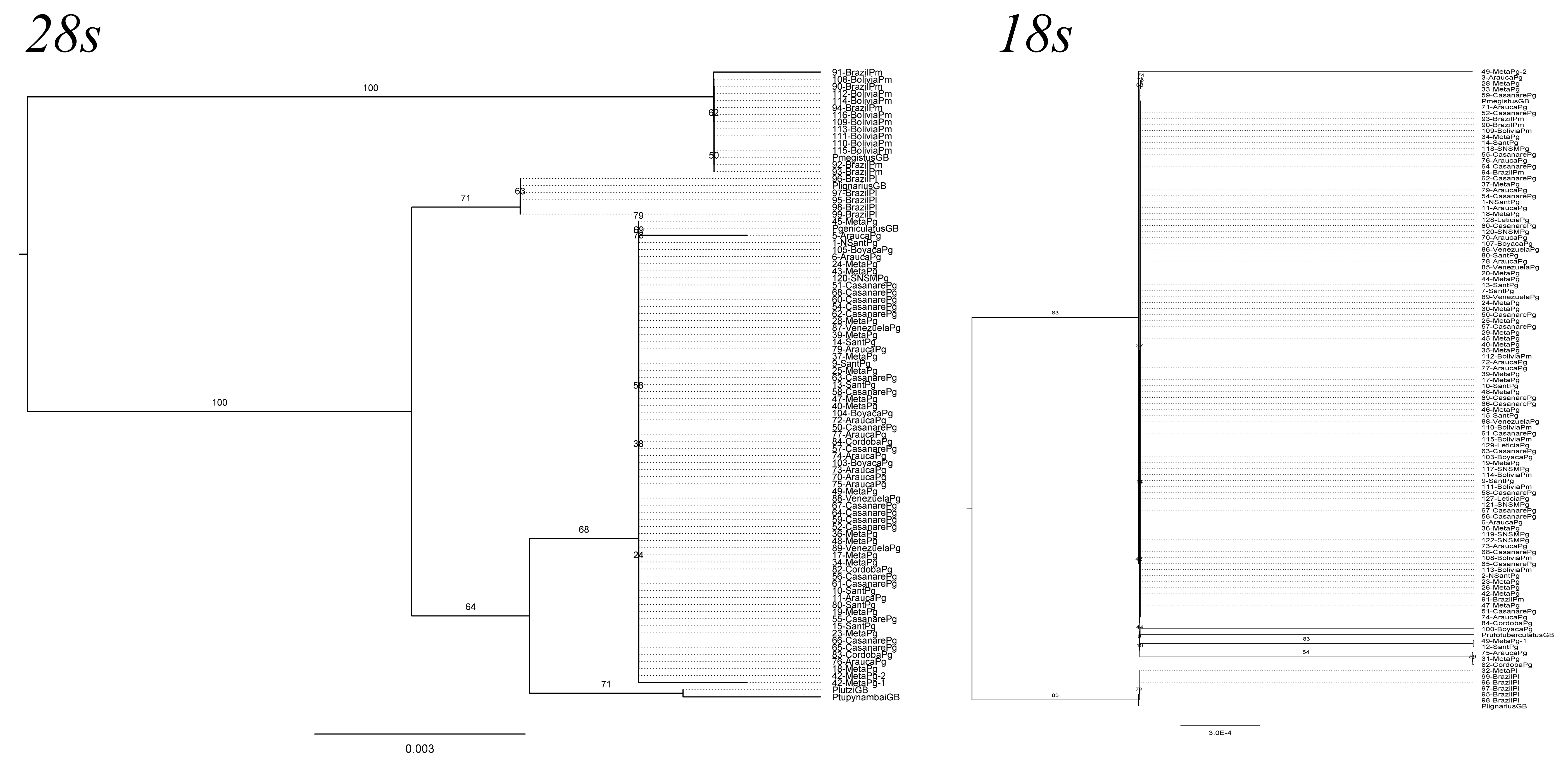

Supplement: S1 Fig — Numbers at the nodes are the bootstrap support values after 10,000 bootstrap replicates. (TIF) [file pone.0223963.s005.tif]
